# Intestinal Microbiota in Patients with Spinal Cord Injury

**DOI:** 10.1371/journal.pone.0145878

**Published:** 2016-01-11

**Authors:** Bilgi Gungor, Emre Adiguzel, Ihsan Gursel, Bilge Yilmaz, Mayda Gursel

**Affiliations:** 1 Department of Molecular Biology and Genetics, Middle East Technical University, Ankara, Turkey; 2 Department of PMR, Gulhane Military Medical Academy, Turkish Armed Forces Rehabilitation Center, Ankara,Turkey; 3 THORLAB, Department of Molecular Biology and Genetics, Bilkent University, Ankara, Turkey; University of Illinois at Chicago, UNITED STATES

## Abstract

Human intestinal flora comprises thousands of bacterial species. Growth and composition of intestinal microbiota is dependent on various parameters, including immune mechanisms, dietary factors and intestinal motility. Patients with spinal cord injury (SCI) frequently display neurogenic bowel dysfunction due to the absence of central nervous system control over the gastrointestinal system. Considering the bowel dysfunction and altered colonic transit time in patients with SCI, we hypothesized the presence of a significant change in the composition of their gut microbiome. The objective of this study was to characterize the gut microbiota in adult SCI patients with different types of bowel dysfunction. We tested our hypothesis on 30 SCI patients (15 upper motor neuron [UMN] bowel syndrome, 15 lower motor neuron [LMN] bowel syndrome) and 10 healthy controls using the 16S rRNA sequencing. Gut microbial patterns were sampled from feces. Independent of study groups, gut microbiota of the participants were dominated by *Blautia*, *Bifidobacterium*, *Faecalibacterium and Ruminococcus*. When we compared all study groups, Roseburia, Pseudobutyrivibrio, Dialister, Marvinbryantia and Megamonas appeared as the genera that were statistically different between groups. In comparison to the healthy group, total bacterial counts of *Pseudobutyrivibrio*, *Dialister and Megamonas* genera were significantly lower in UMN bowel dysfunction group. The total bacterial count of *Marvinbryantia* genus was significantly lower in UMN bowel dysfunction group when compared to the LMN group. Total bacterial counts of *Roseburia*, *Pseudobutyrivibrio and Megamonas* genera were significantly lower in LMN bowel dysfunction group when compared to healthy groups. Our results demonstrate for the first time that butyrate-producing members are specifically reduced in SCI patients when compared to healthy subjects. The results of this study would be of interest since to our knowledge, microbiome-associated studies targeting SCI patients are non-existent and the results might help explain possible implications of gut microbiome in SCI.

## Introduction

Human intestinal tract is colonized by thousands of different genera of bacterial species whose number and genetic content exceed that of the host by a factor of ten and 150-fold, respectively [1]. These commensal microorganisms and their metabolites have substantial effects on the host, modulating the functions of the immune system, [2], the endocrine system, the enteric nervous system [3] and the central nervous system [4]. The growth and the composition of intestinal microbiota are affected by a plethora of factors, including immune mechanisms, dietary factors and intestinal motility. Recent studies have shown that an imbalance of the normal gut microbiota (dysbiosis) is associated with inflammatory bowel diseases [5], irritable bowel syndrome [6] and some other diseases [7].

Patients with spinal cord injury (SCI) commonly have neurogenic bowel dysfunction due to absence of central nervous system control over the gastrointestinal system. Bowel problems can cause major physical and psychological difficulties for these patients [8]. There are two different bowel dysfunction types In SCI: upper motor neuron (UMN) and lower motor neuron (LMN) bowel syndrome [9,10]. The UMN bowel syndrome is associated with increased anal sphincter and colonic wall tonus. Although stool propulsion and reflex mechanisms are preserved due to intact neuronal circuitry between the colon and the spinal cord, the intestinal transit time has been shown to be reduced. These patients often represent with constipation and fecal retention [9]. In contrast, the LMN bowel syndrome or flaccid bowel is associated with loss of reflex mechanisms. Constipation is frequent in this bowel type since stool propulsion is very slow. There is also a high risk for incontinence due to atonic sphincters [11].

Gut is known to be a target organ for various kinds of stress triggered by sepsis, shock, burn, trauma and infection [12]. Considering that SCI is a stressor altering a number of physiological processes, we hypothesized that bowel dysfunction and altered colonic transit time in SCI patients could lead to a significant change in the composition of gut microbiome. The objective of this study was to characterize the gut microbiota in adult SCI patients with different types of bowel dysfunction. Our results demonstrate for the first time that butyrate-producing members are specifically reduced in SCI patients when compared to healthy subjects. These findings could be of interest in defining the probable impacts of microbiome dysbiosis in SCI patients.

## Materials and Methods

### Patients and controls

Subjects were recruited from a national rehabilitation center of SCI clinic and healthy controls (able-bodied) were included from the same hospital employees. Written informed consent form was obtained from all participants and the study protocol was approved by the Kecioren Education and Research Hospital, Ethics Committee (Approval #: B10.4.ISM.4.06.68.49, Date: 13/March/2013). Patients were included if they met the following criteria: 1) neurologically complete (as defined by the American Spinal Injury Association, Atlanta, GA, USA) spinal cord injury (level of injury above T-6) or traumatic cauda equina syndrome occurring 12 or more months prior to the study, 2) 18 years of age or older, 3) traumatic spinal cord injury. Exclusion criteria included use of antibiotics in the past three weeks and incomplete SCI. All patients underwent a general examination and a full American Spinal Injury Association (ASIA) examination before being included in the study. Demographic data, bowel management types, infection frequency and history of antibiotic use was recorded. To exclude probable effects of diet on microbiota, all patients and healthy subjects were fed with standard hospital food 1–3 weeks before stool collection. There were no other diet restrictions. Laboratory staff was blinded to the case-control identification.

### Sample collection and DNA isolation

Gut microbial patterns were determined from stool samples. Patients collected the samples into sterile disposable containers in commode chairs or during a side-lying position in the bed without using enema. Control group also collected samples into the same disposable containers. Faecal samples were frozen and kept at -80°C until use. For isolation of bacterial DNA from stool samples, PowerSoil bacterial DNA extraction kit (MoBio, Carlsbad CA) was used with some modifications. Prior to the isolation protocol described by the manufacturer, each sample was homogenized using a sterile disposable spatula and treated with Lysozyme (10 μg/ml final concentration). Extraction was then continued using the bead-beating method in the presence of lysozyme.

### 16S rRNA gene amplification

The 16S rRNA gene V4 variable region PCR primers 515/806 (515F GTGCCAGCMGCCGCGGTAA and 806R GGACTACVSGGGTATCTAAT) with barcode on the forward primer were used in a 30 cycle PCR using the HotStarTaq Plus Master Mix Kit (Qiagen, USA) under the following conditions: 94°C for 3 minutes, followed by 28 cycles of 94°C for 30 seconds, 53°C for 40 seconds and 72°C for 1 minute, after which a final elongation step at 72°C for 5 minutes was performed. After amplification, PCR products were verified on a 2% agarose gel to determine the success of amplification and the relative intensity of bands. Multiple samples were pooled together in equal proportions based on their molecular weight and DNA concentrations. Pooled samples were purified using calibrated Ampure XP beads. The pooled and purified PCR product was used to prepare the DNA library following Illumina TruSeq DNA library preparation protocol. Sequencing was performed on a MiSeq following the manufacturer’s guidelines. The barcodes and primers were trimmed from the sequences and then short sequences < 200bp were removed from the raw data. The sequences that contained 6bp and bigger homopolymer regions and ambiguous base calls were removed. Sequences were then denoised and chimeras removed. Operational taxonomic units (OTUs) were identified after removal of sequences clustering at 3% divergence (97% similarity). OTUs were then taxonomically grouped and classified using BLASTn tool against a curated GreenGenes database [13] and compiled into each taxonomic level into both “counts” and “percentage” files. Counts files contain the actual number of sequences while the percent files contain the relative (proportion) percentage of sequences within each sample that map to the designated taxonomic classification.

### Bioinformatics and statistical data analyses

Relative abundance of identified organisms were normalized prior to analysis based on maximum read counts per sample. The organisms with low relative frequencies (<%0.1) were filtered. The remaining 100 different types (at the genus level) were used for group comparison analysis. All analyses used the RStudio-software package (v.0.98.1091). Significance of differences in the relative abundances between experiment and control groups were assessed by ANOVA (analysis of variance) method. Group based comparisons between the experiment and control microbial groups with a confidence interval %95 were carried out using Tukey’s Test using the "TukeyHSD" function of the R package “stats”. Hierarchical clustering based on the R package “cluster” was generated using average linkage. Heatmaps used the heatmap.2 function based on R package “gplots”.

## Results

### Patient and control groups

**Fig 1:**
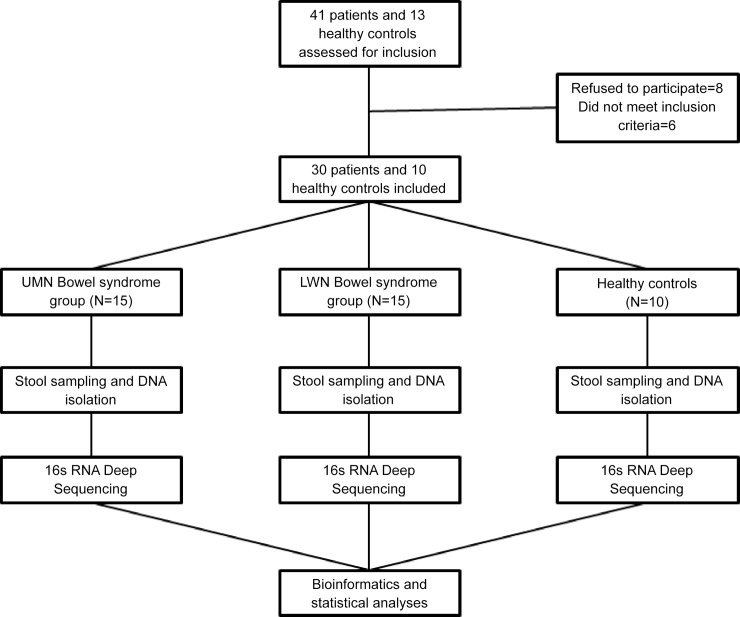
Flow diagram of the study design (UMN: Upper motor neuron, LMN: Lower motor neuron).

Patients were divided into two groups according to their bowel dysfunction types. Fifteen complete spinal cord injury patients (level of injury above T6) were included in the upper motor neuron (UMN) bowel syndrome group whereas 15 patients with cauda equina syndrome were included in the lower motor neuron (LMN) bowel syndrome group (Fig 1). Ten healthy subjects were included in the control group. Characteristics of the participants are shown in Table 1. All of the patients with SCI had neurologically complete injuries (ASIA-A) and all injuries were due to contusion of spinal cord. Levels of injury within the patient groups were as follows: UMN group: C4 (n = 1, 6.7%), C5 (n = 1, 6.7%), C6 (n = 1, 6.7%), C7 (n = 1, 6.7%), T3 (n = 1, 6.7%), T4 (n = 4, 26.7%), T5 (n = 6, 40.0%); LMN group: T12 (n = 5, 33.3%), L1 (n = 9, 60.0%), L2 (n = 1, 6.7%). There was no statistically significant difference between groups in terms of age and gender. Comparisons of etiology and disease interval were performed between UMN and LMN bowel syndrome groups. Bowel management types (Chi-square test, p>0.05) and antibiotic usage (Mann-Whitney U test, p>0.05) were not statistically different between the groups. According to participants’ self-reports, urinary tract infection rates per year were 3.0±1.8 and 2.6±1.8 in UMN bowel syndrome and LMN bowel syndrome groups, respectively. In healthy control group, nobody reported urinary tract infection.

**Table 1:** Demographic characteristics of the participants.

	UMN Bowel Syndrome	LMN Bowel Syndrome	Healthy Controls	p
Age (mean years (s.d.))	35.0 (9.5)	34.0 (8.9)	34.4 (8.0)	>0.05*
Sex (%male;%female)	86.7;13.3	93.3;6.7	100.0;0.0	>0.05**
Months from injury (median, (min-max))	21.0(13.0–105.0)	18.0(13.0–94.0)	NA	>0.05*
Etiology (%)				>0.05**
Motor vehicle collisions	46.6	46.7	NA	
Fall from an elevated height	40.0	26.7	NA	
Gunshot wound	6.7	26.7	NA	
Diving into shallow water	6.7	-	NA	

*Kruskal-Wallis test

**Chi-square test

UMN: Upper motor neuron, LMN: Lower motor neuron, NA: Not applicable.

### Characterization of intestinal microbiota

**Fig 2:**
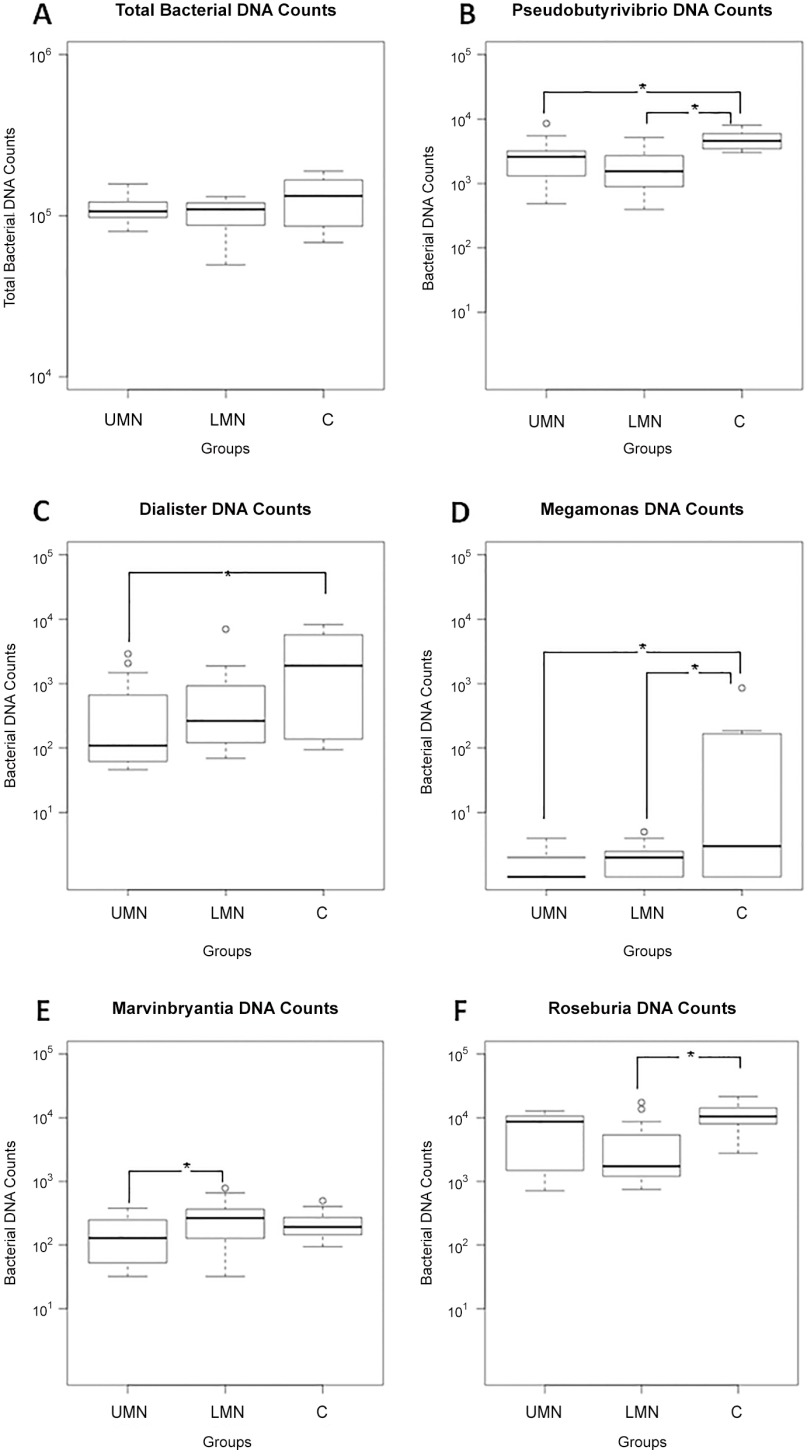
Gut microbiome composition profiles that differed between UMN group (n = 15), LMN group (n = 15), and control group (n = 10). The values represent bacterial DNA counts in gut microbiome. (A) Total bacterial counts; (B) Pseudobutyrivibrio; (C) Dialister; (D) Megamonas; (E) Marvinbryantia; (F) Roseburia. The horizontal lines in the boxplots show median values and the whiskers show the 5–95 percentiles. Values below and above whiskers are represented with dots. UMN: Upper motor neuron; LMN: Lower motor neuron; C:Control; *p<0.05.

**Fig 3:**
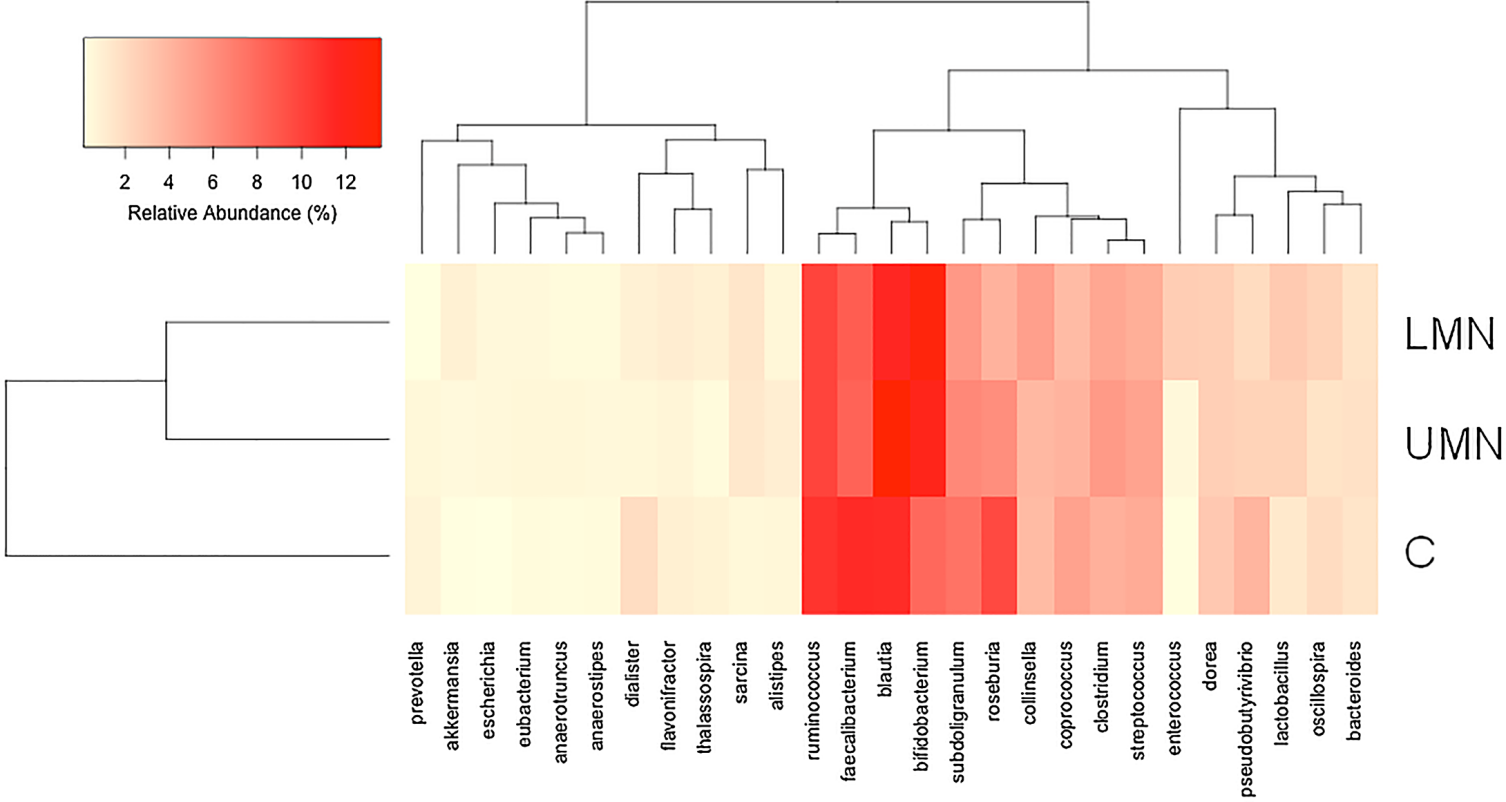
Hierarchical cluster analysis showing the relative percent abundance of each genus in the study groups. UMN: Upper motor neuron group; LMN: Lower motor neuron group; H: Healthy controls.

16S rRNA gene sequences were generated using Illumina's MiSeq platform. Briefly, a total of 4,444,553 reads were obtained with a mean of 111,000 reads per participant. Reads were clustered in OTUs at 97% of identity. Independent of study groups, gut microbiota of the participants were dominated by *Blautia*, *Bifidobacterium*, *Faecalibacterium* and *Ruminococcus*. Total bacterial DNA counts were similar between groups (Fig 2A). Hierarchical cluster analysis shows the relative percent abundances of each genus in different groups (Fig 3, and S1 Fig). The analysis was based on UPGMA (Unweighted Pair Group Method with Arithmetic Mean) method. Compositional dissimilarity between different categories were calculated using the Bray–Curtis dissimilarity method.

### Comparison between study groups

To highlight possible GI flora differences between groups, SCI patients were compared to healthy controls. To exclude age related fecal composition differences, we selected age-matched healthy subjects. Both patient and control groups were on the same diet profile and did not use antibiotics for the last 3-weeks period. When we compared all study groups, *Roseburia*, *Pseudobutyrivibrio*, *Dialister*, *Marvinbryantia* and *Megamonas* appeared as the genera that were statistically different between groups.

### Gut microbiota in UMN bowel dysfunction group

In comparison to the healthy group, total bacterial counts of *Pseudobutyrivibrio*, *Dialister* and *Megamonas* genera were significantly lower in UMN bowel dysfunction group (p = 0.019, p = 0.042 and p = 0.029 respectively, Tukey’s HSD test) (Fig 2B, 2C and 2D respectively). The total bacterial count of *Marvinbryantia* genus was significantly lower in UMN bowel dysfunction group (p = 0.021, Tukey’s HSD test) (Fig 2E) when compared to the LMN group. These results suggest that Pseudobutyrivibrio, a butyrate, lactic acid and formic acid producer [14] and Bacteroides members Dialister and Megamonas that are involved in interactions with the intestine [15] are significantly reduced in UMN bowel dysfunction group.

### Gut microbiota in LMN bowel dysfunction group

Total bacterial count of *Marvinbryantia* genus was significantly higher in LMN bowel dysfunction group (p = 0.021, Tukey’s HSD test) (Fig 2E) when compared to the UMN bowel dysfunction group. Furthermore, total bacterial counts of *Roseburia*, *Pseudobutyrivibrio* and *Megamonas* genera were significantly lower in LMN bowel dysfunction group (p = 0.019, p = 0.002 and p = 0.031 respectively, Tukey’s HSD test) (Fig 2F, 2B and 2D, respectively) when compared to healthy controls. Roseburia and Marvinbryantia belong to the Lachnospiraceae family that degrade complex polysaccharides to short chain fatty acids including acetate, butyrate, and propionate [16]. Our results show that compared to healthy controls, there is a preferential decrease in Marvinbryantia in the UMN group, whereas in the LMN group, Roseburia is decreased. These results suggest that although the identity of the genera that are dysregulated in both groups may differ, the outcome (i.e reduced butyrate production) would be expected to be similar.

## Discussion

In this study we hypothesized that gut microbiome in SCI patients may change according to their bowel dysfunction types and would be different when compared to healthy subjects. We tested our hypothesis on 30 SCI patients and 10 healthy controls using the 16S rRNA sequencing. The results of this study would be of interest since to our knowledge, microbiome-associated studies targeting SCI patients are non-existent and the results might help explain possible implications of gut microbiome dysbiosis in SCI.

Firmicutes and Bacteroides spp. are the most predominant phylum in the gut [17]. They ferment non-digestible polysaccharides and generate metabolites that can be used for energy by the host. Among these, acetate, propionate and butyrate are among the most well characterized single chain fatty acid metabolites that are produced following carbohydrate fermentation in the gut [18]. Butyrate is the most pronounced single chain fatty acid (SCFA) with modulatory effects on epithelial cell growth and differentiation and immune function [18,19]. Of interest, microglia, the resident macrophages of the central nervous system contributes to secondary tissue damage and axonal retraction following spinal cord injury [20,21]. Short chain fatty acids, butyrate in particular, have potent anti-inflammatory effects on macrophages [22,23] and can suppress ongoing inflammation in the CNS [24]. In this context, our findings revealed a significant reduction in butyrate producing phylum members in SCI patients, suggesting that reduced levels of butyrate may contribute to microglia-mediated neurotoxicity in these patients. It is plausible that low butyrate levels may have an impact on long-term recovery after SCI. Of note, glial cells are also known to increase pain hypersensitivity by releasing signaling molecules like multiple pro-inflammatory cytokines [25–28]. Considering microglia mediated neurotoxicity and their contribution to pain sensation, reduced levels of butyrate may be a persistent triggering factor for neuropathic pain.

Gastrointestinal transit times are significantly increased in UMN and LMN bowel syndromes and did not differ from each other [29]. Motility of the upper gastrointestinal tract is mainly modulated by vagal output. The underlying reason(s) accounting for gastrointestinal dysmotility following SCI remains enigmatic considering that the vagal output remains intact in these patients. Our results revealed that Marvinbryantia Spp. is significantly decreased in UMN group in comparison with LMN group. We suspect that such a alteration between the UMN and LMN groups might not be related to bowel motility differences but might imply that the existence of this commensal might be affected by the autonomic nervous system dysfunction in the UMN group. One other explanation to account for this decrease might be a compensation mechanism operating in response to the changes in levels of other commensals. Since total bacterial DNA counts were not significantly different in UMN and LMN groups, this might be a reasonable explanation.

We believe that our findings demonstrating dysbiosis in gut microbiome following SCI could initiate future studies to address unresolved issues such as how the microbiome of patients with SCI affects bidirectional microbiota-gut-brain axis and whether this relates to brain reorganization after SCI. Emerging evidence imply that gut microbiome exerts considerable modulatory effects of on host neural functions and development [3]. Dysbiosis in microbiota can affect the immune system, regulating CNS functions and thus change mood and behavior [30,31].

Immune dysfunction in patients living with SCI is crucial in terms of increased infection tendency. When the effects of microbiome on the development and modulation of immune system are considered, it is likely that a possible contribution of altered microbiome on this immune dysfunction might be expected. In this study we only characterized gut microbiome and did not investigate its impact on the immune system. However, how microbiome alters immune functions in SCI remains an important question and might be investigated in future studies.

A strong point of this study was the inclusion of only complete SCI patients with level of injury above T-6 or cauda equina syndrome. This approach excluded probable confounding effects of thoracic splanchnic nerves on gut functions, and therefore other levels of injuries were not included in this study. Participants were fed with standard hospital food for 1–3 weeks before stool collection. However, whether a longer term (i.e. >2 weeks) dietary recall would be necessary to account for individual diet-associated flora differences could not be determined and remains as a major weakness of this study.

## Supporting Information

S1 Fig Combined data with relative percentages among groups. UMN: Upper motor neuron group (Group A, N = 15); LMN: Lower motor neuron group (Group B, N = 15); and H: Healthy controls (Group C, N = 10).
(XLSX)
